# Purification of the full-length *Xenopus* interphotoreceptor retinoid binding protein and growth of diffraction-quality crystals

**Published:** 2007-12-13

**Authors:** Debashis Ghosh, Jennifer B Griswold, Thomas Bevilacqua, Federico Gonzalez-Fernandez

**Affiliations:** 1 Hauptman-Woodward Medical Research Institute; 2 Pharmacology and Therapeutics, Roswell Park Cancer Institute; 3 Department of Structural Biology, State University of New York at Buffalo; 4 Neuroscience Graduate Program State University of New York at Buffalo; 5 Ross Eye Institute, Departments of Ophthalmology, Pathology, and Biochemistry, State University of New York, Medical Research Service, Veterans Affairs Medical Center, Buffalo, New York

## Abstract

**Purpose:**

Interphotoreceptor retinol-binding protein (IRBP), composed of two or four homologous modules in tandem, plays an important role in retinoid trafficking between the retinal pigmented epithelium, photoreceptors, and Müller cells. The exact nature of this role is not yet clear. Attempts to purify the full-length retinal IRBP to homogeneity for crystallization purposes have largely been unsuccessful, owing primarily to instability and denaturation of the protein at high concentrations in aqueous media.

**Methods:**

A bacterial expression system was used for the production of the recombinant full-length four modules-containing *Xenopus* IRBP (xIRBP; 1197 amino acids; 131 kDa). An optimized purification strategy and the presence of molar excesses of a thiol-based reducing agent yielded highly pure xIRBP in a soluble, stable and active form, free of its fusion partner. Binding of all-trans retinol was characterized by fluorescence spectroscopy monitoring ligand-fluorescence enhancement, quenching of endogenous protein fluorescence, and energy transfer.

**Results:**

We grew the first diffraction-quality crystal of xIRBP. We have gathered diffraction data from these crystals to 2.46 Å resolution, sufficient to yield an atomic model of the tertiary structure of IRBP. Retinol-binding results determined by fluorescence spectroscopy show roughly one retinol-binding site per polypeptide chain.

**Conclusions:**

The binding stoichiometry taken together with modeling data suggest that not all modules are functionally equivalent. Determination of the full-length IRBP structure will be a significant breakthrough in understanding the functional roles of IRBP in the visual cycle. The advances presented here will not only lead to the structure of the full-length IRBP, but will allow us to understand how the modules interact in the function of IRBP. Furthermore, these studies will allow characterization of the ligand-binding site(s) with bound ligand(s).

## Introduction

The functional significance of the module structure of interphotoreceptor retinoid-binding protein (IRBP) remains unknown. IRBP, roughly a 135 kDa (~1200 amino acids) glycoprotein, is composed of four homologous “modules.” Each are approximately 300 amino acids in length. Rods and cones secrete IRBP into subretinal space, where it is the major soluble protein component of the interphotoreceptor matrix (IPM). In the IPM, IRBP is rapidly turned over by a combination of retinal pigmented epithelium (RPE) and photoreceptor endocytosis [1–3]. Although insight into its structure, cellular physiology, and function has been accumulating [4–8], progress is severely hampered by lack of information about its complete three-dimensional structure.

IRBP is positioned to mediate interactions between cells bordering the IPM. IRBP carries visual cycle retinoids in a light-dependent manner, and was originally thought to only solubilize retinoids in the IPM. Its role is rather complex as it functions in four steps of the visual cycle: promoting (1) the removal of all-trans retinol from bleached rod outer segments [9,10], (2) the delivery of all-trans retinol to the RPE [11], (3) the release of 11-cis retinal from the RPE [12–14], and (4) the delivery of 11-cis retinal to the outer segments [14]. The role of IRBP in these steps, although sufficient, may not be strictly necessary, as IRBP-deficient mice (transgenic “knockout” mice) do not show a depressed rate of retinoid exchange between the retina and RPE [15]. The rate of recovery is more rapid than that in controls [16,17]. This could be explained by a buffering activity, a property that has been observed in studies monitoring the exchange of retinoids between lipid vesicles [18]. The importance of IRBP as a buffer may be related to its biochemical activity in preventing oxidative and isomeric degradation of visual cycle retinoids [19]. The mechanism of IRBP’s antioxidant activity is not known. Finally, it should be mentioned that IRBP has been implicated in functions outside of the visual cycle including retinal development (reviewed in [5,8]), and fatty acid binding [20,21]. The emerging picture is that IRBP has multiple functions in the visual cycle, and probably has roles outside the cycle. Understanding the mechanisms involved will require a clearer picture of its structure.

*Xenopus* provides a particularly useful experimental system to study the trafficking, and function of IRBP in the visual cycle and in development. We have, thus, continued to focus on *Xenopus* IRBP (xIRBP; 131 kDa; 1197 amino acids without the signal peptide) as an alternative model for human IRBP, which is 60% identical to xIRBP in amino acid sequence. Furthermore, xIRBP is composed of four modules that are homologous to that of mammalian and avian IRBPs [22,23]. In the current study, we have overcome the problems related to generating IRBP crystals. Here, we extend our previous study [24] by preparing a full-length xIRBP in a soluble form free of its thioredoxin (Trx) fusion partner, establish conditions that allow for concentrating the protein without precipitation, and identify conditions that permit routine growth of diffraction quality crystals. These advances will not only lead to the structure of the full-length IRBP, but will also allow characterization of ligand-binding domain(s) with bound ligand.

## Methods

### Expression of the recombinant full-length *Xenopus* interphotoreceptor retinol-binding protein and purification by Ni-affinity chromatography

Preparation of the clone expressing xIRBP as a thioredoxin fusion protein that contains a His-patch has been previously described [24]. The fusion protein DNA was sub-cloned into pThiohisA vector (Invitrogen, Carlsbad, CA); the bacterial cell used for protein expression was Top10 (Invitrogen). Cell lysate in a binding buffer of 25 mM Tris-HCl, pH 7.5 at 4 °C, containing 200 mM NaCl and 1mM Dithiothreitol (DTT; Inalco, San Luis Obispo, CA) was applied to a pre-equilibrated 5ml HisTrap HP column (GE Healthcare, Piscataway, NJ). All purification columns were run on an Äkta FPLC (GE Healthcare). Protein was eluted over a 20-column volume linear gradient from 0 to 300 mM imidazole in an elution buffer composed of 25 mM Tris-HCl, pH 7.5 at 4 °C, 50 mM NaCl, and 0.5 mM DTT. A major protein peak eluted at 50 mM imidazole. The eluted fractions were pooled, and the protein concentration was determined by measuring the optical density at 280 nm (OD_280_) and by sodium dodecyl sulfate-PAGE (SDS–PAGE) analysis performed with 12% Tris-HCl pre-cast gels (Bio-Rad Laboratories, Hercules, CA). Pooled fractions were diluted such that the imidazole concentration was 20 mM or less. To remove the N-terminal Trx fragment, we added EnterokinaseMax (EK; Invitrogen) in a ratio of 2 U EK to 1 mg protein. After incubation for ~36 h at 4 °C, EK was removed using EK-Away resin (Invitrogen) according to the manufacturer’s direction.

The cleaved xIRBP was then applied to a second 5 ml HisTrap HP column (GE Healthcare) pre-equilibrated with the binding buffer. The same chromatography method was used on a Äkta FPLC (GE Healthcare). Protein was eluted over a 20-column volume linear gradient from 0 to 300 mM imidazole with the following elution buffer: 25 mM Tris-HCl, pH 7.5 at 4 °C, 50 mM NaCl, and 0.5 mM DTT. While a part of the cleaved xIRBP flowed through the column as expected, some bound the column, presumably through the endogenous histidine side chains, and eluted as a major peak at an imidazole concentration of 60 mM. The bound fractions were pooled, and the protein concentration was determined by SDS–PAGE analysis, and absorbance spectroscopy using extinction coefficients calculated from the primary sequence [24]. Proteolysis was prevented by adding 0.4 mM Pefabloc SC (Roche, Indianapolis, IN) to the pooled fractions.

### Gel filtration

The DTT concentration of the protein solution was then adjusted to 1 mM, and resulting solution was concentrated to a final volume of about 6 ml by ultrafiltration using a YM-50 centricon (Millipore, Billerica, MA). The protein was then loaded onto a previously equilibrated Sephacryl S-300HR (S300) column (GE Healthcare), which measured 2.6 × 100 cm. The gel filtration procedure was run on a Äkta FPLC with one column volume isocratic elution using the following binding buffer: 25 mM Tris-HCl, pH 7.5 at 4 °C, 50 mM NaCl, and 1 mM DTT. xIRBP eluted as dimeric as well as monomeric peaks, each of which were pooled and analyzed by SDS–PAGE. The purest xIRBP fractions eluted as a dimer between elution volumes 212 and 250 ml. The procedure yielded 15 to 20 mg of ~97% pure xIRBP from 1.25 L of cell culture.

### Protein assay

Final IRBP-containing fractions were pooled, and protein concentration was determined by OD_280_ and 12% SDS–PAGE analysis.

### N-terminal sequence

The xIRBP band from the SDS–PAGE analysis was blotted onto a PVDF membrane. The band was cut out, dried, and sent to Proseq (Boxford, MA) for sequencing by the Edman degradation method.

### Crystallization

The buffer components of purified xIRBP were adjusted to 50 mM Tris-HCl, pH7.5 at 4 °C, 100 mM NaCl, and 4 mM DTT. The protein solution was then concentrated to12 mg/ml of xIRBP. At the final concentration, 0.5 mM oleic acid was added to the solution, and the mixture was incubated. Crystal screening experiments were conducted with freshly purified xIRBP using the MbClass II Suite commercial screen (Qiagen/Nextal, Montreal, Canada) cocktail solutions. The screening was performed at 23 °C in 24-well sitting drop plates. Crystals initially grew at 23 °C from 0.1M MgCl_2_, 0.1M NaCl, 0.1 M Na HEPES pH 7.5, 12% polyethylene glycol (PEG) 4000, as well as from 0.1M MgCl_2_, 0.1 M NaCl, 0.1M 3-(Cyclohexylamino)-2-hydroxy-1-propanesulfonic acid (CAPSO); pH 9.5, 12% PEG 4000 when the protein and the reservoir (cocktail) solutions were mixed in 1:1 ratio and the resulting droplets were allowed to undergo vapor diffusion against reservoirs in sealed wells. Following optimization of the growth conditions, best diffraction-quality crystals were routinely grown at 23 °C from 0.1M MgCl_2_, 0.1M NaCl, 0.1M Tris-HCl pH 8.0, 14%–22% PEG 4000 in a 3:1 protein:cocktail ratio.

### X-ray diffraction experiment

Initial X-ray diffraction experiments were performed on the in-house R-AXIS IVC image plate and rotating anode X-ray system, with the crystals maintained both at ambient and cryogenic temperatures. X-ray diffraction data collection was performed at the A-1 station of the Cornell High Energy Synchrotron Source (CHESS), Ithaca, New York. The crystals were cooled at cryogenic temperature by plunging them into liquid nitrogen and maintaining them in a stream of liquid nitrogen during data collection. Glycerol was added to the crystallization droplets to adjust the final glycerol concentration to 40%–50%. This liquid mixture served as the cryoprotectant for the crystals. The diffraction images were recorded on an ADSC Quantum-210 Charge Coupled Device (CCD) detector. The diffraction data were processed with HKL2000 [25] and MOSFLM [26] routines.

### Ligand-binding assays by fluorescence spectroscopy

The binding of all-*trans* retinol to *Xenopus* IRBP was characterized in titrations using a DM 45 scanning spectrofluorimeter (Online Instrument Systems, Inc., Bogart, Georgia) [27]. Titrations monitoring enhancement of retinol fluorescence, quenching of the intrinsic protein fluorescence, and energy transfer were performed as previously described [28]. Enhancement of retinol fluorescence was followed by monitoring the increase in retinol fluorescence (excitation: 330 nm; emission: 480 nm) compared to a fluorescence-matched solution of N-acetyl-L-tryptophanamide [29]. The concentration of the N-acetyl-L-tryptophanamide solution was adjusted to match the fluorescence of the apo-IRBP solution. The fluorescence of all-*trans* retinol in the presence of N-acetyl-L-tryptophanamide was subtracted from that in the presence of xIRBP. Assays following quenching of protein fluorescence used an excitation wavelength of 280 nm and an emission wavelength of 340 nm. In these experiments the inner filter effect was accounted for by graphical correction [30]. Energy transfer assays monitored retinol fluorescence at 480 nm (excitation: 280 nm). The dissociation constant (*K_d_*) and number of binding sites (*N*) was determined by nonlinear least square fit to a binding equation that assumed a single type of noninteracting site(s) [31].

## Results

### Stability and integrity of purified *Xenopus* interphotoreceptor retinol-binding protein

**Figure 1:**
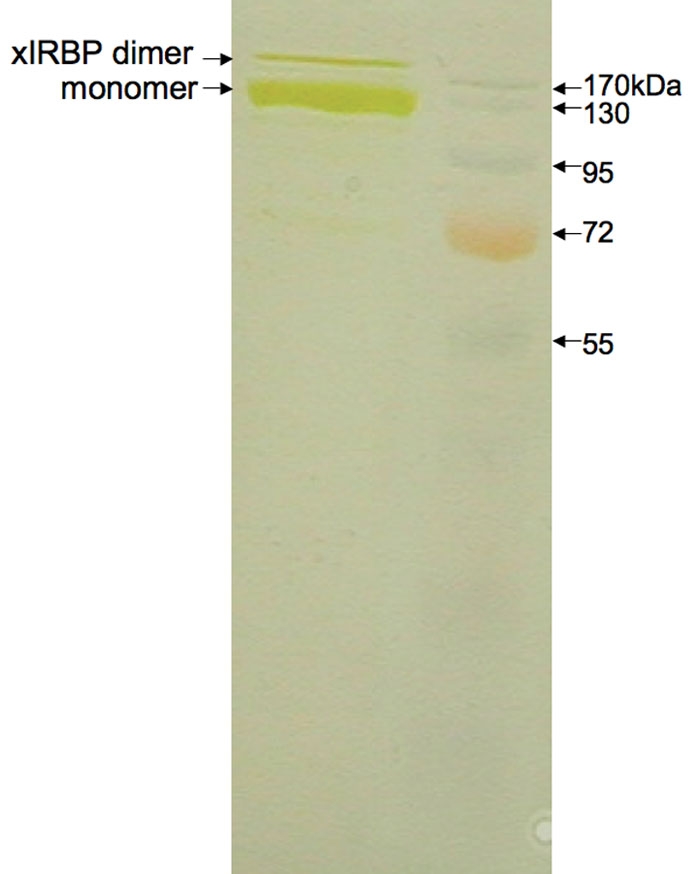
Gel electrophoresis (SDS–PAGE; silver stained) analysis of the purified four-module containing *Xenopus* interphotoreceptor retinol-binding protein. The major band corresponds to monomeric *Xenopus* interphotoreceptor retinol-binding protein (xIRBP) molecular weight of 131 kDa. The minor higher molecular weight band represents dimeric form of the protein, which may or may not be an artifact of the analysis itself.

The expession and purification procedure described in the previous section yielded 15 to 20 mg of ~97% pure xIRBP from 1.25 L of cell culture. A gel electrophoresis of the final product is shown in Figure 1. In contrast to previous reports of xIRBP instability at high concentrations [32], the purified xIRBP, free of its fusion partner Trx, maintained its integrity, high solubility, and ligand-binding ability for weeks at 4 °C at a concentration of ~10 mg/ml. The enhanced protein stability and sustained solubility were partially attributable to the presence of molar excess of DTT in solution. Furthermore, the absence of n-octyl-β-D-glucopyranoside or any other detergent was probably a stabilizing factor as well, since for soluble proteins, detergents could add to instability promoting gradual unfolding. Thus, under the reducing conditions of the purification experiments, highly pure xIRBP maintained its naturally folded stable tertiary structure, and, therefore, was fully soluble and functionally active for a prolonged period of time.

### N-terminus sequence

The N-terminal sequence for the full-length recombinant xIRBP was unambiguously determined to be G-L-G-D-P-F-Q-P-S-L. The actual N-terminal sequence of xIRBP is F-Q-P-S-L… The first part of the determined sequence, namely G-L-G-D-P, belonged to the vector pThioHisA (Invitrogen) that was included in the cloning sequence of xIRBP. This result, along with the molecular weight of the protein band (Figure 1), confirmed the identity of the purified protein to be the full-length recombinant xIRBP, free of its fusion partner Trx.

### Binding assays with all-*trans* retinol

**Figure 2:**
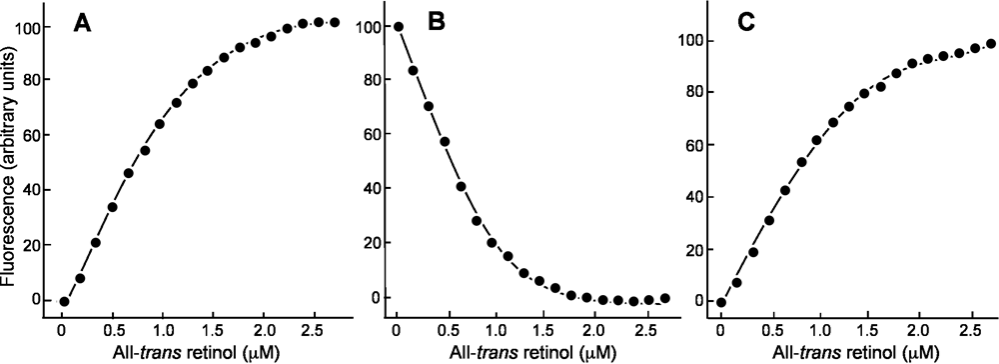
Fluorescence titrations are shown of *Xenopus* interphotoreceptor retinol-binding protein (xIRBP) binding all-trans retinol. The concentration of xIRBP was 0.89 μM in each panel. **A:** The titration of IRBP with all-trans retinol in this panel is followed by monitoring the increase in retinol fluorescence compared to a fluorescence matched solution of N-acetyl-L-tryptophanamide (excitation: 330 nm; emission: 480 nm). **B:** The titration shown follows the quenching of intrinsic protein fluorescence by bound retinol (excitation: 280 nm; emission: 340 nm). **C:** The titration follows ligand binding by monitoring energy transfer (excitation: 280 nm; emission: 480 nm).

Binding curves for fluorescence studies for binding of all-*trans* retinol are shown in Figure 2. For the increase in retinol fluorescence measurements (excitation: 330 nm; emission: 480 nm), the number of binding sites per molecule of protein (*n*) was 1.30±0.04 with *K_d_*=0.15±0.03 μM. Titration monitoring quenching of tryptophan fluorescence by bound retinol (excitation: 280 nm; emission: 340 nm) yielded binding parameters n=1.32±0.06 and *K_d_*=0.16 ± 0.04 μM. Finally, the calculated binding parameters were n=1.05 ± 0.03 and *K_d_*=0.06± 0.01 μM for titration monitoring of energy transfer.

The retinol-binding data compare well with the data from groups purifying the protein in the presence of DTT, albeit at concentrations lower than the present work of Chen et al. [33]. Older studies, which apparently did not include a reducing agent in the purification of xIRBP, tended to obtain values for N and *K_d_* of roughly one site and 1 μM, respectively [11,34].

### Crystallization and X-ray diffraction

**Figure 3:**
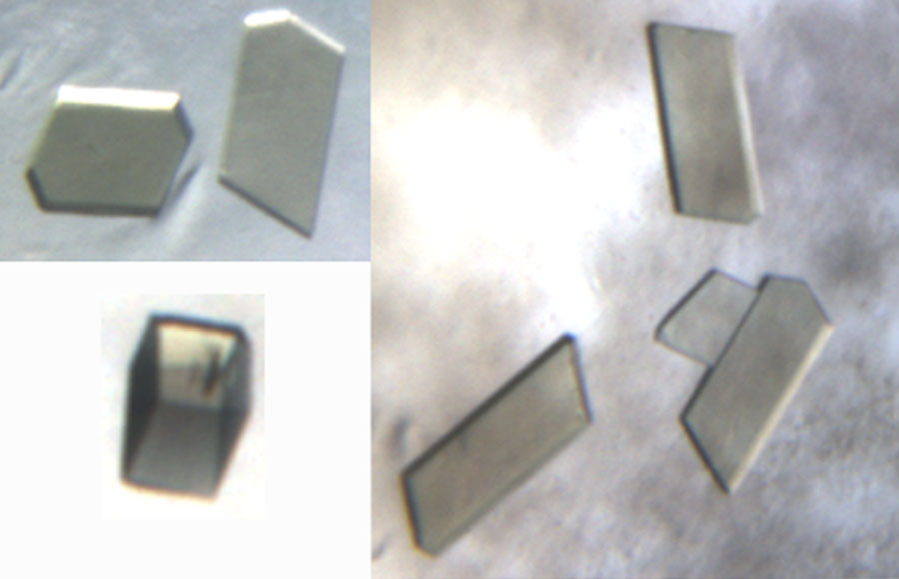
Photomicrographs of *Xenopus* interphotoreceptor retinol-binding protein crystals grown in the presence of oleic acid. The crystals were typically about 0.2 mm in the longest dimension.

**Figure 4:**
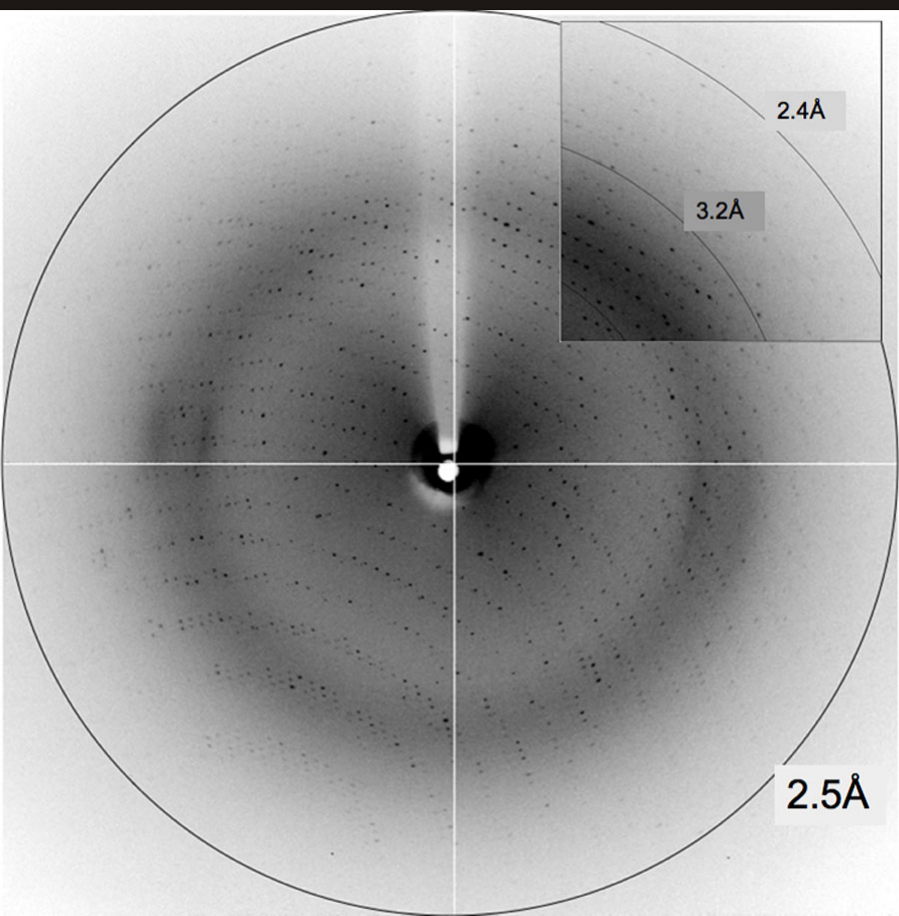
An X-ray diffraction pattern from a *Xenopus* interphotoreceptor retinol-binding protein crystal recorded at the A-1 station of the Cornell High Energy Synchrotron Source. The detector was at a distance 250 mm; the wavelength, the oscillation angle and the exposure time were 0.978 Å, 1°, and 15 s, respectively. The right upper corner segment is shown in higher contrast to demonstrate the limiting resolution of diffraction of ~2.45 Å.

Single crystals of xIRBP appeared in 5–10 days at 23 °C and continued to grow for weeks. The crystals had two different morphologies: plate-like and parallelepiped-shaped. The largest and best-formed crystals were typically about 0.2 mm in the longest dimension. Figure 3 is a photomicrograph of crystals of xIRBP that grew from a buffer containing 0.1 M MgCl2, 0.1 M NaCl, 0.1 M Tris-HCl (pH8.0) with 20%–22% PEG 4000 as the precipitant, at 2:1 and 3:1 protein to reservoir ratios. A typical diffraction image recorded with synchrotron X-rays at the CHESS is shown in Figure 4. Crystals of both morphologies had the same space group and showed similar diffraction characteristics, typically to a resolution of about 2.45 Å. The space group was P2_1_ and the cell parameters were a=93.53 Å, b=134.04 Å, c=121.29 Å, β=109.8°. There were two 131 kDa polypeptide chains in the asymmetric unit, giving rise to a specific volume of 2.65 Å^3^/Da, which was within the range for protein crystals [35]. From one crystal a total of 180 diffraction images, each of 1° oscillation and 15 s exposure, were recorded on a CCD detector yielding 318,878 total observations for 99,902 unique reflections. The data was 99.3% complete to 2.46 Å resolution, with an R_merge_ of 0.099. The overall intensity to standard deviation ratio is 16.9 and that in the highest resolution shell (2.55–2.46 Å) is 3.6.

## Discussion

IRBP is the only known retinoid-binding protein that possesses a multiple domain structure. However, the exact nature of its role has remained a mystery, owing partially to the lack of structural insights into the nature of binding pockets, and correlation of the functional properties with structural details. Biochemical and homology modeling studies suggest that the ligand-binding domains in each module are not equivalent, and that the modules interact with each other [24]. Thus, understanding IRBP’s complex function in the visual cycle will require a detailed knowledge of the structural and functional relationships of its modules. The structure of the xIRBP module 2 [36] has provided some clues to that effect. Nonetheless, since a functional full-length *Xenopus* and mammalian IRBP molecule contains four homologous modules, these structures in the ligand-bound form are likely to yield more comprehensive understanding of the structure-function relationship. Our effort to investigate the structure of full-length xIRBP in complex with fatty acids, and retinoids is a step toward achieving that goal.

Determining the structure of IRBP has proven difficult. First, IRBP is large (~131 kDa), and undergoes ligand-dependent conformational changes [37]. This together with its glycosylation properties [38] could contribute to molecular and dynamical heterogeneities. Purification of tens of milligrams of xIRBP to high homogeneity and in pristine form as well as successful growth of the high diffraction-quality crystals demonstrate that by using the recombinant protein, a bacterial expression system, and optimized purification strategies, we have been able to eliminate the heterogeneities and instabilities of the past. The use of a thiol-based reducing agent in prevention of denaturation is significant since IRBPs contain several cysteine residues [39]. Homology modeling of modules 1, 3, and 4 of xIRBP after the module 2 structure suggests that many, if not all, of its seven cysteines do not participate in disulfide formation and remain as free –SH groups exposed to the solvent. Interestingly, of the 10 cysteines in bovine IRBP, eight were experimentally determined to have free –SH groups [39]. Our data for bovine IRBP indicate that molecules aggregate, unfold, and denature irreversibly by cross-linking through free sulfhydryl groups (unpublished results), which may result in previously observed limited solubility [32]. Although some enhancement of solubility can be achieved by the addition of detergents, such as n-octyl-β-D-glucopyranoside, slow precipitation through denaturation of the protein cannot be prevented [32]. Although the function of IRBP’s free –SH groups are unknown, it is plausible that they may have a role in IRBP’s possible antioxidant activities for the protection of reduced retinol molecules in the visual cycle [19].

The all-*trans* retinol binding results derived with the structurally pristine xIRBP from three separate experiments, namely (1) enhancement in retinol fluorescence, (2) quenching of intrinsic protein fluorescence, and (3) titration monitoring of energy transfer, are internally consistent and suggest roughly one retinol binding site per four-module full-length molecule with a dissociation constant of about 0.1μM. This data can be interpreted as the retinol-binding site being restricted to one of the four modules, or to an intramodule site defined by multiple modules, consistent with the notion that not all four modules are functionally equivalent as the homology modeling results indicated. Interestingly, homology modeling identified the presence of a highly hydrophobic cavity, the so-called site I, suitable for retinol binding in modules 1 and 2, and predicted that this site in modules 3 and 4 may not exist [24]. Some differences between the two retinol-binding sites in modules 1 and 2 were identified. It is likely that only one of these site I sites actually binds retinol, and the other is unavailable owing to the quaternary association of the four modules. The second type of sites in each module, the so-called site II, was more open, hydrophilic, and predicted to be lower affinity retinol or possibly fatty acid binding sites. Interestingly, the current xIRBP crystals were grown in the presence of 0.5 mM oleic acid, prompting us to speculate that binding of ligands to the specific sites may provide additional dynamical stability necessary for the growth of diffraction-quality crystals. Efforts to grow xIRBP crystals in the presence of retinol and other ligands are now in progress. Elucidation of the nature and number of the ligand-binding sites and their implications in overall IRBP function must await the determination of the crystal structures of some of these complexes.
